# Heparin-Mimicking Polymer-Based In Vitro Platform Recapitulates In Vivo Muscle Atrophy Phenotypes

**DOI:** 10.3390/ijms22052488

**Published:** 2021-03-02

**Authors:** Hyunbum Kim, Ji Hoon Jeong, Mona Fendereski, Hyo-Shin Lee, Da Yeon Kang, Sung Sik Hur, Jhaleh Amirian, Yunhye Kim, Nghia Thi Pham, Nayoung Suh, Nathaniel Suk-Yeon Hwang, Seongho Ryu, Jeong Kyo Yoon, Yongsung Hwang

**Affiliations:** 1 Soonchunhyang Institute of Medi-Bio Science (SIMS), Soonchunhyang University, Cheonan-si 31151, Korea; tiggerhy@snu.ac.kr (H.K.); jjh2020@sch.ac.kr (J.H.J.); mona.fendereski@gmail.com (M.F.); hyo7093@sch.ac.kr (H.-S.L.); sstahur@sch.ac.kr (S.S.H.); yhkim@sch.ac.kr (Y.K.); ptnghia@sch.ac.kr (N.T.P.); ryu@sch.ac.kr (S.R.); 2 School of Chemical and Biological Engineering, Institute of Chemical Processes, Seoul National University, Seoul 08826, Korea; nshwang@snu.ac.kr; 3 Department of Integrated Biomedical Science, Soonchunhyang University, Asan-si 31538, Korea; 4 Department of Pharmaceutical Engineering, Soonchunhyang University, Asan-si 31538, Korea; gubin15@naver.com (D.Y.K.); nysuh@sch.ac.kr (N.S.); 5 Institute of Tissue Regeneration, Soonchunhyang University, Asan-si 31538, Korea; jalehamirian@gmail.com

**Keywords:** synthetic mimic of heparin, poly(sodium-4-styrenesulfonate), myoblast, myogenic differentiation, fusion, focal adhesion kinase (FAK)

## Abstract

The cell–cell/cell–matrix interactions between myoblasts and their extracellular microenvironment have been shown to play a crucial role in the regulation of in vitro myogenic differentiation and in vivo skeletal muscle regeneration. In this study, by harnessing the heparin-mimicking polymer, poly(sodium-4-styrenesulfonate) (PSS), which has a negatively charged surface, we engineered an in vitro cell culture platform for the purpose of recapitulating in vivo muscle atrophy-like phenotypes. Our initial findings showed that heparin-mimicking moieties inhibited the fusion of mononucleated myoblasts into multinucleated myotubes, as indicated by the decreased gene and protein expression levels of myogenic factors, myotube fusion-related markers, and focal adhesion kinase (FAK). We further elucidated the underlying molecular mechanism via transcriptome analyses, observing that the insulin/PI3K/mTOR and Wnt signaling pathways were significantly downregulated by heparin-mimicking moieties through the inhibition of FAK/Cav3. Taken together, the easy-to-adapt heparin-mimicking polymer-based in vitro cell culture platform could be an attractive platform for potential applications in drug screening, providing clear readouts of changes in insulin/PI3K/mTOR and Wnt signaling pathways.

## 1. Introduction

Skeletal muscle, the largest organ in the human body, comprises approximately 40% of the total body mass of healthy individuals [1]. Due to the hierarchical structure of skeletal muscles and the intrinsic structure–function relationship between the skeletal muscle extracellular matrix (ECM) and skeletal muscle stem cells (skMSCs), cell–matrix interactions within skeletal muscle have been extensively investigated [2,3,4]. While bioengineered models for enhancing skeletal muscle function have been extensively developed and explored, there have been fewer advancements in the development of highly functional muscle atrophy-specific organ-on-a-chip models compared to in vivo muscle injury models [5,6]. 

Cell fusion and cell–matrix interactions within the microenvironment are considered key cellular processes that govern the formation and repair of skeletal muscle, in turn maintaining skeletal muscle homeostasis [7,8]. During this process, activated skMSCs fuse to form multinucleated myotubes, forming parallel bundle structures, which eventually comprise functional skeletal muscle tissue [9,10]. Therefore, it is critical to understand the molecular mechanism underlying myogenic commitment, fusion, and the maturation of activated skMSCs into fully functional myofibers, including the various signaling pathways involved. One way to explore these is through the use of an in vitro skeletal muscle-like drug screening platform [11].

The microenvironment surrounding skMSCs is known to trigger cellular responses, which alter various aspects of cellular function, including the cell adhesion, spreading, proliferation, and differentiation of skMSCs [12,13,14]. Similarly to the multitude of signaling cues, such as growth factors, small molecules, and hormones, direct regulation of stem cell fate also occurs in response to matrix stiffness, and thus the topographical features of cell culture substrates are considered important factors for the stimulation of signal transduction and subsequent cell lineage determination [15,16,17,18]. Molecular dynamics within focal adhesions have been shown to regulate cellular adhesion through integrins and the ECM, with these biomechanical signals being transmitted in a bidirectional manner [19,20]. In addition, physicochemical interactions between cells and their microenvironment consistently provoke intercellular signaling, in turn modulating gene expression and diverse signal transduction [21,22,23,24]. 

Previous studies have demonstrated the important roles of basic fibroblast growth factor (bFGF), a heparin-binding protein, in the regulation of skMSC proliferation and differentiation as well as its contribution to skeletal muscle homeostasis [25]. In addition, cell surface-bound heparin and heparin sulfate proteoglycans (HSPGs) have been proven as necessary for the modulation of EGF signaling and the determination of terminal myogenesis [26,27]. Previously, Sangaj et al. performed molecular docking simulations and reported that the soluble form of synthetic mimics of heparin, poly(sodium-4-styrenesulfonate) (PSS), could be thermodynamically favorable for binding bFGF in a manner equivalent to that of native heparin. Thus, the soluble form of PSS could sequester bFGF to promote myogenic differentiation [28]. 

In this study, we investigated whether a heparin-mimicking polymer-based cell culture substrate with a negatively charged surface could be further utilized as an in vitro platform for the regulation of cell adhesion-mediated myoblast functions, aiming to recapitulate in vivo muscle atrophy-like phenotypes. Additionally, through transcriptome profiling of cells cultured on various matrices, we further elucidated the underlying molecular mechanisms inducing the inhibition of mononucleated myoblast fusion into multinucleated myotubes.

## 2. Results

### 2.1. Characterization of the Physical Properties of GelMA-Based Hydrogels

**Figure 1:**
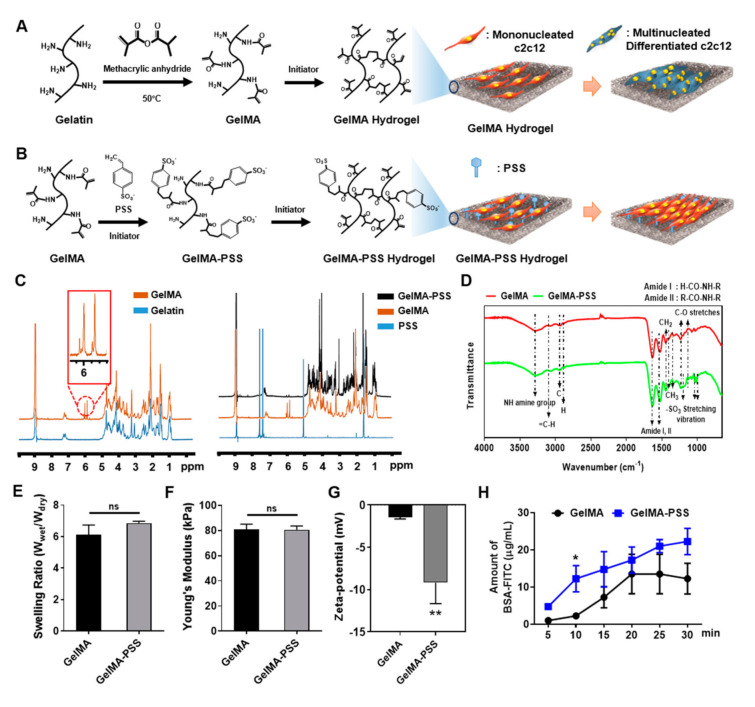
Schematic illustration and characterization of GelMA and GelMA-PSS hydrogels. (**A**,**B**) Synthesis scheme for GelMA and GelMA-PSS hydrogels, respectively. (**C**) ^1^H-Nuclear magnetic resonance (NMR) spectra of synthetic polymer. (**D**) Fourier transform infrared spectroscopy (FTIR) analysis of GelMA and GelMA-PSS. (**E**–**G**) Characterization of various physical properties including swelling ratio, Young’s modulus, and zeta-potential of GelMA and GelMA-PSS. (**H**) Quantification of BSA absorption using FITC-BSA on GelMA and GelMA-PSS hydrogels. Data are presented as the mean ± SD. * *p* < 0.05; ** *p* < 0.01; ns, not significant.

Previously, numerous studies have demonstrated the potential roles of heparin-mimicking polymers in various biological applications in tissue engineering and regenerative medicine approaches [29,30]. In order to synthesize covalently photo-crosslinkable gelatin-based hydrogels, gelatin was initially methacrylated (GelMA), and GelMA was further co-polymerized with the synthetic heparin mimic, PSS, hereafter termed GelMA-PSS (Figure 1A,B). As shown in Figure 1C, the ^1^H nuclear magnetic resonance (^1^H-NMR) spectra revealed the characteristics peaks corresponding to vinyl protons observed at 5.978 and 5.845 ppm, indicative of the successful binding of methacrylate groups into gelatin. Furthermore, the ^1^H-NMR spectra at 1.783 ppm peak indicated that PSS was successfully co-polymerized with GelMA. To further confirm the synthesis of GelMA-PSS hydrogel, Fourier transform infrared spectroscopy (FTIR) analysis was performed on a pre-polymerized solution containing GelMA and PSS (Figure 1D). The transmittance of the gelatin solution alone showed an NH amine group and a C–O stretch at 1242 and 1148 cm^–1^, respectively. The percent transmittance of GelMA-specific peaks was reduced when the PSS solution volume was increased. Additionally, the FTIR spectrum of GelMA-PSS revealed an –SO_3_ stretch at 1186, 1043, and 1014 cm^–1^, exhibiting the successful co-polymerization of PSS into GelMA. 

Next, the GelMA and GelMA-PSS hydrogels were polymerized using redox initiators, and various mechanical properties, such as swelling ratios, Young’s modulus, and zeta-potential, were characterized. As shown in Figure 1E,F, the swelling ratios and Young’s modulus of both GelMA and GelMA-PSS were not significantly different, indicating that the incorporation of PSS into GelMA does not affect the mechanical properties of GelMA hydrogels. We further characterized the PSS-mediated negative charge distribution and its contribution to protein adsorption on the hydrogel surface via zeta-potential analysis and a fluorescein isothiocyanate (FITC)-conjugated bovine serum albumin (FITC-BSA) binding assay (Figure 1G,H). Zeta-potential analysis demonstrated that the anionic charge distribution of the GelMA hydrogel was −1.05 to −1.67 mV, whereas that of the GelMA-PSS hydrogel was −5.34 to −13.8 mV, suggesting that integration of the sulfonate functional groups of PSS into the GelMA hydrogel results in an additive anionic charge on its hydrogel surface. Additionally, the FITC-BSA binding assay revealed that due to the higher degree of negative charges on GelMA-PSS hydrogels, theses could adsorb a higher amount of FITC-BSA when compared to GelMA hydrogels at all time points. 

### 2.2. Effect of Heparin-Mimicking Matrix-Based Cues on the Initial Cell Adhesion and Proliferation of Myoblasts

**Figure 2:**
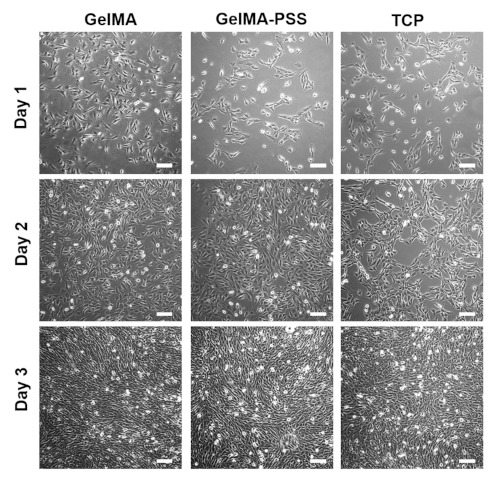
Phase-contrast images of cells adhered onto GelMA (**left column**), GelMA-PSS (**middle column**), and tissue culture plate (TCP) (**right column**) as a function of cell culture time up to day 3. Scale bar = 200 µm.

To assess whether heparin-mimicking matrix-based cues could support cell adhesion and proliferation of murine myoblasts, c2c12 cells were seeded onto tissue culture plates (TCPs), GelMA, and GelMA-PSS hydrogels. As shown in Figure 2, our initial observation found that cells were able to adhere to all cell culture substrates and showed highly similar spindle cell morphology, evident by phase-contrast microscopy. Further, adhered c2c12 cells cultured on all groups reached confluence within 72 h, confirming that the anionic surface introduced by PSS did not have any detrimental effects on cell adhesion, spreading, and growth, which is in accordance with our previous report [30].

### 2.3. Inhibition of Myogenesis by Heparin-Mimicking Matrix-Based Cues 

**Figure 3:**
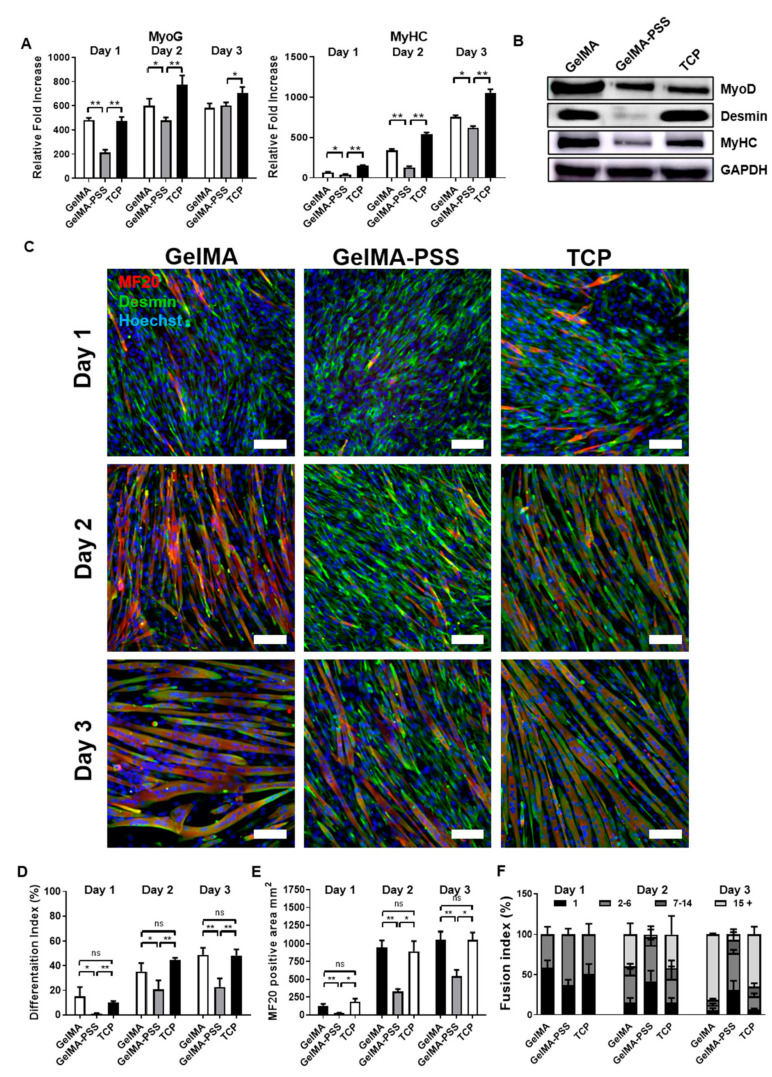
Myogenic potential of cells cultured on GelMA, GelMA-PSS and TCP. (**A**) Gene expression profiles of cells cultured on GelMA, GelMA-PSS, and TCP for 1–3 days in a myogenic induction medium. (**B**) Myogenic protein expression of cells cultured on GelMA, GelMA-PSS, and TCP for 2 days in a myogenic induction medium, as evaluated by Western blot. (**C**) Immunofluorescence staining of MF20 (red) and desmin (green) and nuclei (blue) in cells cultured in a myogenic induction medium on GelMA, GelMA-PSS, and TCP for 1–3 days. Scale bar = 100 µm. (**D**–**F**) Estimated differentiation and fusion indices of cells cultured in a myogenic induction medium on GelMA, GelMA-PSS, and TCP for 1–3 days (for GelMA, *n* = 203, 184, 286 at day 1,2,3; for GelMA-PSS, *n* = 405, 267, 273 at day 1,2,3; for TCP, *n* = 281, 523, 341, respectively). Data are presented as the mean ± SD, * *p* < 0.05; ** *p* < 0.01; ns, not significant.

Given the importance of matrix-based cues, including matrix stiffness, protein immobilization, and topographical features of cell culture substrates, with regard to cell adhesion, differentiation, as well as myoblast fusion [17,24,31], we examined whether heparin-mimicking matrix-based cues presented on the surface of GelMA-PSS hydrogels could affect the myogenic differentiation of c2c12 cells. Upon full confluence, myogenic differentiation was induced for up to 3 days using TCP, GelMA, and GelMA-PSS as cell culture substrates. Thereafter, myogenic potential was evaluated via qualitative polymerase chain reaction (qPCR), Western blot, and immunofluorescence staining. As shown in Figure 3A, the gene expression profiles of various myogenic markers revealed that cells cultured on both TCP and GelMA hydrogels could undergo robust myogenic differentiation, confirmed by upregulation of the late muscle-specific gene expression of *MyoG* and *MyHC* in a culture time-dependent manner. In contrast, cells cultured on GelMA-PSS hydrogels showed significantly decreased late myogenic marker expression when compared to the TCP and GelMA hydrogel groups. To further corroborate gene expression profiles, we evaluated the protein expression in cells cultured on TCP, GelMA, and GelMA-PSS hydrogels via Western blot analyses. Similarly to the qPCR results, cells cultured on TCP and GelMA hydrogels exhibited elevated protein levels of myogenic markers, such as MyoD, desmin, and MyHC. In contrast, cells cultured on GelMA-PSS produced significantly lower levels of the muscle-specific proteins desmin and MyHC (Figure 3B). 

Next, to determine the degree of myogenic differentiation as a function of culture time, c2c12 cells were cultured on TCP, GelMA, and GelMA-PSS hydrogels and induced to undergo myogenesis for 3 days. Their differentiation potential at each time point was immunohistologically assessed. At the early time point (day 1), cells cultured on both TCP and GelMA hydrogels showed that a few cells were stained positive for desmin, a muscle-specific intermediate filament, and MF20, a sarcomeric myosin heavy chain. Although these cells were initially mononucleated myoblasts on day 1, they were successfully fused to form multinucleated myotubes during days 2–3 (Figure 3C, left and right panels). On the other hand, cells cultured with heparin-mimicking matrix-based GelMA-PSS hydrogels were unable to form MF20-positive myoblasts on day 1 and underwent limited fusion of mononucleated myoblasts into multinucleated myotubes (Figure 3C, middle panel). 

To further validate results from the immunofluorescence staining, we quantified the extent of myogenic differentiation by calculating the differentiation index, determined by the ratio of MF20-positive cells to the total number of cells, as well as by the MF20-positive cellular area. Analyses for differentiation index and MF20-positive cellular area indicated that cells cultured on GelMA-PSS hydrogels had a significantly lower number of MF20-positive myoblasts with undoubtedly smaller myotube sizes (Figure 3D,E), when compared to cells cultured on TCP and GelMA hydrogels. In addition to the differentiation index, we also calculated the fusion index, defined as the ratio of myotubes having either 1, 2–6, 7–14, or >15 nuclei to the total number of MF20-positive cells. Fusion index calculation surprisingly revealed that cells cultured with heparin-mimicking matrix-based cues failed to facilitate fusion of mononucleated myoblasts into matured multinucleated myotubes (>15 nuclei, approximately 3%) until day 3 (Figure 3F). In contrast, during the 3 days of myogenic differentiation, cells cultured on TCP and GelMA hydrogels were able to fuse, forming fully matured multinucleated myotubes, and their fusion indices (>15 nuclei) dramatically increased as a function of culture time (~80% at day 3). No significant differences in MF20-positive area, differentiation, and fusion indices were observed between TCP and GelMA hydrogels. Taken together, these results suggest that the incorporation of PSS inhibits both the gene and protein expression of muscle-specific markers. More importantly, PSS culture restricts the fusion of mononucleated myoblasts into multinucleated myotubes through the cell-to-heparin-mimicking matrix interaction. 

### 2.4. Transcriptional Profiling Reveals the Suppression of Myogenesis-Related Genes by Heparin-Mimicking Polymers

**Figure 4:**
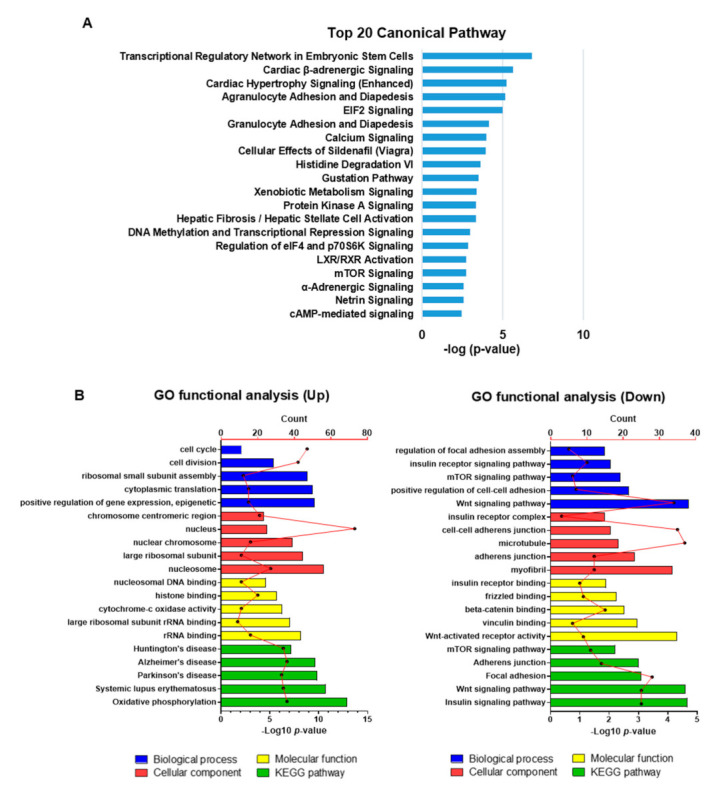
Transcriptome analysis. (**A**) Top 20 canonical pathways commonly enriched in cells cultured on GelMA-PSS. (**B**) Top 5 significantly enriched GO terms functional analysis data based on differentially expressed genes (DEGs) in cells cultured on GelMA-PSS compared to GelMA and TCP. Biological process (Blue), cellular component (red), molecular function (yellow), and KEGG pathways (green).

To elucidate the molecular mechanisms underlying the inhibition of myogenesis by heparin-mimicking matrix-based cues, we performed RNA sequencing (RNA-seq) analysis of cells cultured in myogenic induction medium on TCP, GelMA, and GelMA-PSS hydrogels for 2 days in order to identify differentially expressed genes (DEGs) with an expression change greater than 1.5-fold. After obtaining statistically significance DEGs (*p < 0.05) with an activation z-score of >2 as a cut-off, we performed pathway analysis of all differentially expressed transcripts using Ingenuity Pathway Analysis (IPA, www.ingenuity.com). As shown in Figure 4A, the canonical mTOR pathway, as an important signaling pathway in skeletal muscle development and myogenic differentiation [32], was downregulated in cells cultured with heparin-mimicking matrix-based cues. Furthermore, we performed comparative function and network analyses, observing a list of diseases and cellular functions related to myogenesis, which indicated significant phenotypic changes in cells cultured on GelMA-PSS hydrogels, when compared to those cultured on both GelMA and TCP (Table 1). The top diseases and biofunctions identified by IPA revealed that cells cultured on GelMA-PSS hydrogels exhibited inferior myogenic phenotypes, including down-regulation in formation, quality, quantity, and contractility of the muscle itself or myoblasts, as well as the inhibition of cell fusion for the formation of multinucleated functional myotubes through cell–cell contact, assembly, and formation of intercellular junctions, and cell aggregation. 

Based on DEG functional annotations, gene ontology (GO) functional enrichment analyses, including biological process (BP), cellular component (CC), and molecular function (MF), were carried out to further understand the signaling pathways modulating myoblast differentiation. The top five upregulated and downregulated BP, CC, and MF terms in cells cultured with heparin-mimicking matrix-based cues when compared to those cultured on TCP and GelMA hydrogels are presented in Figure 4B,C. Upregulated GO terms were associated with the cell cycle and division-associated enrichment. On the other hand, downregulated GO terms of cells cultured with heparin-mimicking matrix-based cues when compared to cells cultured on TCP and GelMA hydrogels revealed a downregulation of insulin, Wnt and mTOR signaling pathways, cell–cell adherens junction, myofibril formation, and cell–matrix interaction (vinculin binding), known to be critical in myogenic differentiation as well as in the maturation of myoblasts and skMSCs [32,33,34]. Kyoto Encyclopedia of Genes and Genomes (KEGG) pathway analysis revealed upregulated oxidative phosphorylation (OXPHOS) in cells cultured with heparin-mimicking matrix-based cues along with the upregulation of degenerative disease-associated pathways, which is in accordance with a previous study suggesting that OXPHOS is required at early myogenic commitment, rather than the maturation stage [35]. Similarly to the GO functional enrichment analysis, KEGG pathway analysis revealed that the insulin, Wnt, and mTOR signaling pathways as well as focal adhesions were significantly downregulated in cells cultured with heparin-mimicking matrix-based cues as opposed to those cultured on TCP and GelMA hydrogels. 

### 2.5. Validation of DEGs Using qPCR and Western Blot Analyses

**Figure 5:**
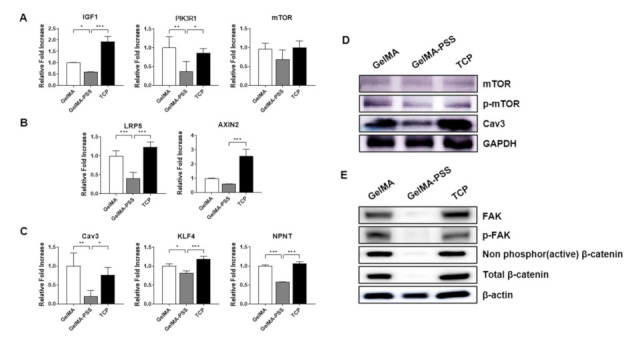
Validation of RNA-seq data via qPCR and Western blot analyses. Gene and protein expression profiles of cells cultured in a myogenic induction medium on GelMA, GelMA-PSS, and TCP for 2 days; (**A**) insulin/PI3K/mTOR signaling pathway, (**B**) Wnt signaling pathway, (**C**) myotube fusion-related markers. Data are presented as the mean ± SD, * *p* < 0.05; ** *p* < 0.01; *** *p* < 0.001; ns, not significant. (**D**) mTOR phosphorylation and Cav protein expression, (**E**) phosphorylated FAK and active β-catenin protein expression.

Having demonstrated the inhibition of myogenic differentiation in cells grown on GelMA-PSS hydrogels, we next sought to validate the findings from the RNA-seq analysis, which indicated that myogenesis was inhibited through the suppression of insulin/PI3K/mTOR and Wnt signaling pathways, as well as through the modulation of focal adhesions. As shown in Figure 5, the cells cultured with heparin-mimicking matrix-based cues exhibited significantly lower expression of various genes associated with the insulin/PI3K/mTOR and Wnt signaling pathways, such as *IGF1*, *RIK3R1*, *mTOR*, Cav3, *LRP5*, and *Axin2*. Furthermore, we found that *KLF4* and its transcriptional target, nephronectin (*NPNT*), were downregulated in cell cultured on GelMA-PSS hydrogels when compared to those grown on TCP and GelMA hydrogels. Western blot analyses (Figure 5B,C) revealed that cells cultured with heparin-mimicking matrix-based cues showed significantly lower protein expression of p-mTOR, Cav3, as well as active and total β-catenin. In an accordance with a previous report demonstrating the important role of focal adhesion kinase (FAK) signaling in myoblast fusion, we observed that the levels of total and phosphorylated FAK, associated with the regulation of integrin-mediated cell adhesion and intracellular signal transduction in the PI3K/AKT/mTOR pathway [36], were significantly suppressed in cells cultured with heparin-mimicking matrix-based cues. 

### 2.6. Validation of the In Vitro Muscle Injury Screening Platform in Comparison to an In Vivo Muscle Injury Model

**Figure 6:**
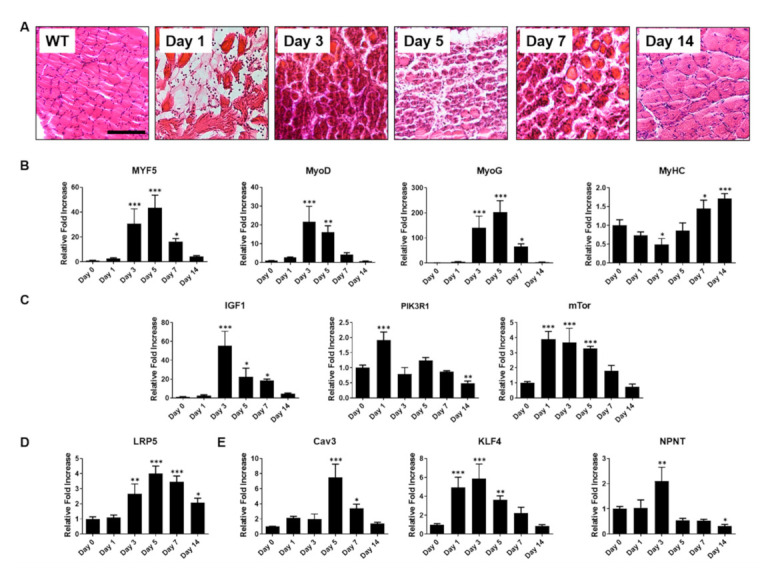
Barium chloride (BaCl_2_)-induced muscle injury model. (**A**) Histological evaluation of barium chloride (BaCl_2_)-injured TA muscles using H&E staining. Scale bar = 100 µm. Gene expression profiles of (**B**) myogenic markers during muscle regeneration, (**C**) insulin/PI3K/mTOR signaling pathways, (**D**) Wnt signaling pathway, (**E**) myotube fusion-related markers. Data are presented as the mean ± SD, * *p* < 0.05; ** *p* < 0.01; *** *p* < 0.001 of six biological replicates for each time point.

To validate the applicability of proof-of-concept that our in vitro heparin-mimicking polymer-based cell culture platform could successfully recapitulate an in vivo muscle injury model, we induced muscle injury in 8-week-old C57BL/6J mice using barium chloride. Tibialis anterior (TA) muscles treated with barium chloride were harvested at different time points up to 14 days post-injury to evaluate whether muscle injury-activated signaling pathways would resemble the in vitro phenotypes of cells cultured with heparin-mimicking matrix-based cues. Non-injured TA muscles served as a control. As shown in Figure 6A, hematoxylin and eosin staining revealed that injection of barium chloride into TA muscles initially caused necrosis of damaged skeletal muscle fibers, as evident by center-located nuclei in degenerating muscle fibers, and a decrease in fiber diameters. Furthermore, at day 5 post-injury, degenerating muscle fibers exhibited a significant muscle atrophy and excessive connective tissue formation within damaged muscle fibers, and finally, damaged muscle fibers were fully regenerated after 14 days. 

Since damaged muscle fibers exhibited typical muscle atrophy phenotypes upon barium chloride-induced muscle injury, we determined the expression of various muscle-specific genes, including those encoding early myogenic markers such as Myf5, MyoD, and MyoG, all of which were significantly elevated during the degeneration processes. In contrast, the late myogenic marker MyHC was considerably upregulated at the later stage of regeneration (Figure 6B). For a comprehensive comparison with cells cultured on GelMA-PSS, we also evaluated genes involved in the fusion and maturation of myoblasts into multinucleated myotubes, namely insulin/PI3K/mTOR and Wnt signaling pathway-related genes. As shown in Figure 6C,D, DEGs identified and confirmed by RNA-seq and qPCR analyses, were upregulated during muscle regeneration. Similarly to previous in vitro results, *KLF4* and *NPNT* genes were upregulated during muscle regeneration, whereas cells cultured on our in vitro platform suppressed their expression, thus mirroring muscle atrophy phenotypes rather than muscle regeneration. 

Taken together, the comparison to the in vivo barium chloride-induced muscle injury model supported the utility of heparin-mimicking matrix-based cues, suggesting that the in vitro platform could simulate muscle atrophy phenotypes by stimulating various pathological alterations of signaling pathways, characteristic of the muscle degeneration process. 

## 3. Discussion

There has been increasing interest in the development of disease-specific in vitro organ-on-a-chip platforms for the accurate assessment of small molecule drugs, including toxicity tests and pharmacokinetics, by recapitulating cell–cell and cell–matrix interactions within tissue-specific microenvironments [11,37,38]. We previously developed a heparin-mimicking polymer-based cell culture substrate to support the long-term expansion and self-renewal of human pluripotent stem cells (hPSCs) while maintaining their pluripotency [30]. In this study, by harnessing a heparin-mimicking polymer with a negatively charged surface due to the presence of sulfonate groups, we engineered an in vitro cell culture platform to facilitate the cell–matrix interaction-mediated inhibition of myoblast function. Thereafter, we sought to recapitulate in vivo muscle atrophy-like phenotypes in cells cultured on our platform. 

Heparin and HSPGs on the cellular surface of myoblasts have previously been shown to bind bFGF with high affinity, promoting the self-renewal of skMSCs [39]. Recently, we harnessed a bFGF-immobilized matrix as a synthetic cell culture substrate to support the cell adhesion and proliferation of skMSCs while maintaining their self-renewal capacity during in vitro expansion [24]. Similarly, we elucidated the roles of various physicochemical cues of a heparin-mimicking anionic polymer PSS, including functional groups, matrix stiffness, surface roughness, and hydrophilicity, with regard to the long-term self-renewal of hPSCs. The presence of negatively charged sulfonate groups in PSS enabled the adsorption of various ECM proteins and bFGF [30]. In our current study, despite their similar physical properties, including swelling ability and matrix stiffness, GelMA-PSS and GelMA hydrogels elicited different cellular responses. These discrepancies in the inhibition of myoblast fusion and terminal differentiation into multinucleated myotubes could be attributed to the presence of negatively charged sulfonate groups in heparin-mimicking polymers (GelMA-PSS hydrogels). Therefore, it is possible that the bioactivity of bFGF bound to sulfonate groups on the GelMA-PSS hydrogel surface through electrostatic interactions can be stabilized without undergoing denaturation and enzymatic degradation, as previously reported [29,40,41], resulting in suppression of the myogenic differentiation of adhered cells. On the other hand, previous studies have demonstrated that the soluble heparin in a culture medium could sequester the bFGF from microenvironment surrounding myoblasts, leading to the induction of terminal differentiation into myocytes [42,43]. Similarly to the pro-myogenic effect of soluble heparin on the myoblast function, exogenous supplement of soluble heparin-mimicking polymers have been shown to downregulate the mitogen activated extracellular regulated signaling kinase (MAPK/ERK) pathway by withdrawing bFGF away from FGFR in a concentration-dependent manner [28,44]. However, it is still unclear whether heparin-mimicking polymers in our study would preferentially regulate the modulation of bFGF stability over sequestering the bFGF. 

Another possibility is the presence of negatively charged sulfonate groups in heparin-mimicking polymers, which could sophisticatedly modulate important signaling pathways involved in muscle homeostasis through cell–matrix interactions [36]. Although our initial findings indicated that there were no significant differences in the initial cell adhesion and proliferation of cells cultured on various cell culture substrates such as GelMA, GelMA-PSS, and TCP (Figure 2), only heparin-mimicking matrix-based cues inhibited the fusion of mononucleated myoblasts into multinucleated myotubes, resulting in the suppression of myogenic differentiation (Figure 3). To distinguish the molecular signatures of cells cultured on heparin-mimicking matrix-based cues, RNA-seq analysis and qPCR validation revealed that the insulin/PI3K/mTOR and Wnt signaling pathways were greatly downregulated (Figure 4 and Figure 5A,B,E), both being tightly associated with skeletal muscle development and myogenic differentiation [45,46]. Furthermore, similarities between our in vitro platform and the in vivo barium chloride-induced muscle injury model were validated, including the suppression of insulin/PI3K/mTOR and Wnt signaling (Figure 6). 

Insulin/PI3K/mTOR signaling has been extensively investigated as a key pathway in skeletal muscle development and regeneration [45,47]. Risson et al. reported that muscle-specific mTOR knockout mice exhibited significant myopathic phenotypes and decreased dystrophin expression, indicative of the crucial role of mTOR in the maintenance of muscle function and metabolism [48]. Cong et al. recently reported that the IGF–Akt–mTOR signaling pathway activated via insulin receptor substrate 1 (IRS1), known as a critical regulator for IGF signaling, could promote myogenic differentiation of myoblasts as well as skeletal muscle regeneration [49]. Furthermore, the Wnt signaling pathway is known to regulate various cellular functions, including cell proliferation, polarity, and cell fate determination, through membrane proteins LRP5/6 and Frizzled, which orchestrated together as receptors for Wnt ligands in the canonical Wnt pathway, in turn regulating myogenic differentiation and skeletal muscle homeostasis [33,50]. 

Over the past decades, studies have shown that the formation of multinucleated myotubes through mononucleated myoblast fusion is an event during myogenic differentiation and skeletal muscle formation [51]. Therefore, we assessed myotube fusion-associated markers, including *Cav3, KLF4,* and *NPNT*, as well as the levels of cell adhesion proteins such as focal adhesion kinase (FAK) and its phosphorylated form (p-FAK) (Figure 4 and Figure 5C–E). Fluck et al. previously reported that FAK was highly expressed during the in vitro fusion of myoblasts into myotubes, and its expression was increased in newly formed skeletal muscles [52]. Similarly, Quach et al. and other research groups have confirmed the crucial role of FAK and its subsequent regulation of Cav3 and β1D integrin during the fusion and maturation of myoblasts into functional myotubes [36,53]. In particular, FAK was proposed as a key regulator of myoblast fusion, whose inhibition lead to the suppression of Cav3 and β1D integrin, resulting in impaired in vivo skeletal muscle regeneration. Sunadome et al. demonstrated that overexpression of KLF2/4 in MEK inhibitor-treated myoblasts could restore their ability to form fused myotubes and identified NPNT as a key factor for myoblast fusion [54]. In agreement with these previous studies, we observed that FAK, Cav3, and NPNT were significantly decreased at both gene and protein levels, confirmed by in vitro as well as in vivo results. Cells cultured on our GelMA-PSS platform resembled the myogenically restricted phonotypes of the barium chloride-induced muscle atrophy phenotypes in vivo. Therefore, the current findings suggest that cells on GelMA-PSS hydrogels could interact with heparin-mimicking moieties and thus exhibit decreased expression of pFAK and Cav3, leading to impaired myoblast fusion. 

In conclusion, our proof-of-concept study confirmed that the GelMA-PSS hydrogel-based in vitro platform recapitulated in vivo muscle atrophy-like phenotypes, as confirmed by the downregulation of key signaling pathways in cells cultured on heparin-mimicking matrix. Our results suggest that heparin-mimicking matrix-based cues determine focal adhesions and cell–cell interactions of cells at the cell–matrix interface through subtle changes in modulation of protein adsorption and bFGF stabilization, as previously reported [30,41]. In turn, the fusion of myoblasts into matured myotubes and the formation of cell–cell/intercellular junctions were inhibited, recapitulating the initial muscle atrophy phenotypes observed during in vivo muscle injury-induced degeneration of skeletal muscles (Figure 4 and Table 1). Therefore, our in vitro platform may serve as a promising muscle injury model for drug screening and toxicity tests.

## 4. Materials and Methods

### 4.1. Synthesis of Methacrylated Gelatin 

Methacrylated gelatin (GelMA) was synthesized according to a previously described method [55]. Briefly, 10 g of gelatin (cat# G1890, Sigma-Aldrich, St. Louis, MO, USA) was dissolved in 100 mL of phosphate-buffered saline (PBS) solution at 60 °C under constant stirring. Then, 8 mL of methacrylic anhydride (cat# 276685, Sigma-Aldrich, St. Louis, MO, USA) was added dropwise to the gelatin solution while stirring vigorously for 2 h. The reaction mixture was mixed with preheated PBS at 60 °C and kept for 15 min. The resulting solution was dialyzed in deionized water at 52 °C for 7 days using a dialysis membrane (MWCO, approximately 12–14 kDa, Spectrum Laboratories, Gardena, CA, USA). Finally, the solution was filtered, lyophilized, and stored at −20 °C. After synthesis, the products were analyzed via ^1^H-NMR by dissolving in deuterium oxide (D2O; Sigma-Aldrich, Saint Louis, MO, USA).

### 4.2. Preparation of GelMA-Based hydrogels 

GelMA-based hydrogels were synthesized as follows: GelMA was dissolved in PBS at a final concentration of 15% *w*/*v* with or without a heparin-mimicking polymer PSS (cat# 94904, Sigma-Aldrich, St. Louis, MO, USA) at a final concentration of 1% *w*/*v*. Ammonium persulfate (APS, cat# 161-0700, Bioshop, Burlington, ON, Canada) and N,N,N′,N′-tetramethylethylenediamine (TEMED, cat# 161-0800, Bioshop, Burlington, ON, Canada) were added to this precursor solution at a final concentration of 0.4% w/v and 0.3% *w*/*v*, respectively. The reaction mixtures, including GelMA and GelMA-co-PSS, hereafter referred to as GelMA-PSS, were polymerized in Bio-Rad 1-mm-spacer glass plates at room temperature. Finally, polymerized hydrogels were punched into 24-well plates, sterilized with 70% ethanol, and washed with fresh PBS for 48 h prior to cell culture experiments. The rinsed hydrogels were incubated in growth medium (high glucose DMEM with 2 mM L-glutamine and 50 units/mL penicillin/streptomycin) containing 10% fetal bovine serum (Corning, Oneonta, NY, USA) overnight before seeding the cells.

### 4.3. Fourier Transform Infrared Spectroscopy (FTIR) 

The FTIR spectra of gelatin (powder), GelMA (lyophilized powder), and GelMA-PSS (lyophilized powder) were analyzed using an FTIR spectrometer (Spectrum 100, Perkin Elmer) with a spectral width ranging from 500 to 4000 cm^–1^ at a spectral resolution of 4 cm^–1^. IR peaks corresponding to the methacrylation of gelatin and copolymerization of PSS into GelMA were analyzed.

### 4.4. Swelling Ratio Measurement

The swelling ratio of each hydrogel construct was measured using the gravimetric method. Briefly, hydrogels were swollen in PBS for 24 h to reach equilibrium, and their wet weights were determined as an equilibrium-swollen state (*W_wet_*). The weighed hydrogels were then lyophilized to measure dry weight (*W_dry_*). The swelling ratios of each hydrogel were calculated according to the following equation:

$$\mathrm{swelling}\;\mathrm{ratio}\;(\%)\;=\;\frac{Wwet}{Wdry}\;\times \;100\%$$

### 4.5. Characterization of Young’s Modulus

To measure the Young’s modulus of hydrogel constructs, hydrogels were equilibrated in PBS for 24 h, and their dimensions, such as height and diameter, were measured prior to the compression test. Compression tests were performed using a universal testing machine (EZ-Test EZ-SX, Shimadzu, Kyoto, Japan) equipped with a 500 N load cell, and samples were compressed up to 30% strain at a strain rate of 1 mm/min. The Young’s modulus was calculated from the linear region of the stress–strain curve (0–10% strain). 

### 4.6. Zeta-Potential Measurement

The synthesized GelMA and GelMA-PSS hydrogel samples were lyophilized, freeze-milled, and dispersed in ultrapure distilled water by sonication. Prepared samples were subjected to ζ-potential measurement at room temperature with a ZetasizerNano ZS (Malvern Instruments Ltd., Malvern, UK). All measurements were performed in triplicate. 

### 4.7. Protein Adsorption Assay Using FITC-BSA

The amount of protein adsorbed onto the GelMA and GelMA-PSS hydrogels was quantified by measuring the amount of fluorescein isothiocyanate-conjugated bovine serum albumin (FITC-BSA, A23015, Thermo Fisher Scientific, Waltham, MA, USA) following a previously reported method [56]. Briefly, hydrogels were incubated with 100 μg/mL of FITC-BSA solution in a dark room for 5, 10, 15, 20, 25, and 30 min. Then, each supernatant solution was transferred to a flat-bottom 96-well plate to measure the fluorescence at 488 nm, and the amount of FITC-BSA in the supernatant was used to calculate the amount of FITC-BSA in hydrogels. The adsorption was calculated from a standard curve generated by a series of known concentrations of FITC-BSA.

### 4.8. Cell Culture 

Prior to myogenic differentiation, undifferentiated murine myoblast c2c12 cells were seeded into the TCP, GelMA, and GelMA-PSS hydrogels at an initial seeding density of 5 × 10^3^ cells/cm^2^ in growth medium containing high-glucose Dulbecco’s modified Eagle’s medium (DMEM; Gibco-BRL, Waltham, MA, USA) supplemented with 10% *v/v* fetal bovine serum (FBS; Gibco-BRL, Waltham, MA, USA), 1% L-glutamine (200 mM, Gibco-BRL, Waltham, MA, USA), and 1% *v/v* penicillin–streptomycin (10,000 U/mL of penicillin and 10,000 g/mL of streptomycin, Gibco-BRL, Waltham, MA, USA) under a 5% CO_2_ atmosphere at 37 °C. When cells reached 80% confluence, the growth medium was replaced with myogenic induction medium containing DMEM supplemented with 2% *v*/*v* horse serum (Gibco-BRL, Waltham, MA, USA) and 1% *v/v* penicillin–streptomycin (Gibco-BRL, Waltham, MA, USA) to induce myogenic differentiation. 

### 4.9. Immunocytochemistry

Cells cultured on TCP, GelMA, and GelMA-PSS were fixed with 4% paraformaldehyde (PFA) for 10 min at room temperature, washed in PBS, blocked with 3% (*w/v*) bovine serum albumin in PBS, and permeabilized with 0.1% (*v/v*) Triton X-100 for 1 h at room temperature. Samples were incubated with primary antibodies (diluted in 1% BSA in PBS), including rabbit anti-Desmin (1:200, Abcam, Cambridge, UK) and mouse anti-MyHC (MF-20) (1:200, Developmental Studies Hybridoma Bank, Iowa City, IA, USA), overnight at 4 °C, washed 3 times in fresh PBS, and incubated with secondary antibodies, including anti-rabbit Alexa 488 (1:200, Thermo Fisher Scientific, Waltham, MA, USA) (diluted in 1% BSA in PBS), anti-mouse Alexa 546 (1:200, Thermo Fisher Scientific, Waltham, MA, USA), as well as with an anti-phalloidin (1:200, Thermo Fisher Scientific, Waltham, MA, USA) antibody for 1 h at room temperature. The nuclei were stained with Hoechst 33342 (2 mg/mL; Thermo Fisher Scientific, Waltham, MA, USA) for 10 min at room temperature. All fluorescence images were acquired by using an inverted microscope (Eclipse Ti-U, Nikon, Tokyo, Japan) at the Soonchunhyang Biomedical Research Core-Facility of the Korea Basic Science Institute (KBSI).

### 4.10. Histochemistry

Barium chloride (BaCl_2_)-injured TA muscle samples were fixed with 4% paraformaldehyde (PFA) for 24 h, gradually dehydrated in ethanol, and embedded in paraffin. The paraffin-embedded samples were processed into 10 µm-thick sections to be used for staining. Briefly, paraffin-embedded sections were de-paraffinized with CitriSolv, rehydrated with a graded series of ethanol, and washed with fresh distilled water. For histopathological analysis, sections were stained with hematoxylin (Mayer’s modified; Abcam, Cambridge, UK) and eosin (Sigma-Aldrich, St. Louis, MO, USA). All bright-field images were acquired using an inverted microscope (Eclipse Ti-U, Nikon, Tokyo, Japan) at the Soonchunhyang Biomedical Research Core-Facility of the KBSI.

### 4.11. Image Analysis 

To quantify the degree of myogenic differentiation of murine myoblast c2c12 cells, myosin heavy chain-positive cellular area, differentiation, and fusion indices were calculated using NIH ImageJ software, as previously described [18,57]. The differentiation index was determined as the ratio of MF20-positive cells to the total number of cells, and the fusion index was calculated as the ratio of myotubes having either 1, 2–6, 7–14, or >15 nuclei to the total number of MF20-positive cells. At least 1000 nuclei per group were analyzed from three random fields of view at each time point. 

### 4.12. qPCR 

Total RNA was isolated using TRIzol reagent (Invitrogen, Carlsbad, CA, USA) and reverse transcribed using ReverTra Ace qPCR RT Master Mix with gDNA Remover (Toyobo, Osaka, Japan) according to the manufacturer’s protocols. qPCR was carried out using the SYBR Green Real-time PCR Master Mix (Toyobo, Osaka, Japan) on a StepOnePlus Real-Time PCR System (Applied Biosystems, Foster City, CA, USA) at the Soonchunhyang Biomedical Research Core-Facility of the KBSI. The expression levels of target genes were normalized against glyceraldehyde-3-phosphate dehydrogenase (GAPDH), the ΔCt values were calculated as Ct_target_ − Ct_GAPDH_, and relative fold inductions were calculated using the 2^−^^ΔΔCt^ method. The primer sequences used in this study are presented in Table 2.

### 4.13. RNA-Seq and Data Analysis

Total RNA was isolated using TRIzol reagent (Invitrogen, Carlsbad, CA, USA), and samples were sent to Macrogen (Macrogen Inc., Seoul, Korea) for library preparation and sequencing. Library preparation was performed for transcriptome sequencing using the TruSeq RNA Sample Prep Kit v2 (Illumina, San Diego, CA, USA) following the manufacturer’s instructions. Each library was sequenced using the HiSeq 2500 platform (Illumina, San Diego, CA, USA), and over 25 million 101 bp strand-specific paired-end reads were obtained per sample. DEGs were determined using fragments per kilobase of transcript per million mapped reads (FPKM) values with a change greater than 2-fold and *p* < 0.05 (GelMa-PSS vs. TCP/GelMA). GO analyses of biological function, cellular component, molecular function, and KEGG pathway were carried out using the bioinformatics resource DAVID tool, which provides relevant GO terms associated with DEGs (https://david.ncifcrf.gov/ (accessed on 2 March 2021)). Additionally, canonical pathway and functional network analyses were performed using IPA software (version 42012434, Qiagen, Hilden, Germany).

### 4.14. Western Blot

Cells were lysed in protein extraction solution (RIPA, Elpis Biotech, Daejeon, Korea) with protease and phosphatase inhibitors (Sigma-Aldrich, Saint Louis, MO, USA). Samples were centrifuged at 15,000 rcf and at 4 °C. The protein concentration was determined via Bradford assay. Gel electrophoresis was performed using 6% *w/v* or 12% *w/v* polyacrylamide gels at 100 V, based on the molecular weight of proteins. Proteins were transferred to a polyvinylidene fluoride (PVDF) blotting membrane (GE Healthcare, Chicago, IL, USA) at 350 mA, blocked with 5% skim milk in 1X Tris-buffered saline containing Tween-20 (TBS-T) (pH 7.5), and probed with primary antibodies against GAPDH (2118L, Cell Signaling Technology, Danvers, MA, USA), MyoD (sc-377460, Santa Cruz, Dallas, TX, USA), Desmin (ab8592, Abcam, Cambridge, UK), mTOR (2972S, Cell Signaling Technology, Danvers, MA, USA), p-mTOR (2971S, Cell Signaling Technology, Danvers, MA, USA), Caveolin-3 (ab2912, Abcam, Cambridge, UK), β-catenin (9562, Cell Signaling Technology, Danvers, MA, USA), non-phosphorylated (active) β-catenin (8814, Cell Signaling Technology, Danvers, MA, USA), phospho-FAK (Tyr397) (cat# 700255, Thermo Fisher Scientific, Waltham, MA, USA), FAK (sc-1688, Santa Cruz, Dallas, TX, USA), and β-actin (A5441, Sigma Aldrich, Waltham, MA, USA) diluted at 1:1000, overnight at 4 °C with gentle agitation. Membranes were incubated with horseradish peroxidase-conjugated secondary antibodies (Bio-Rad, Hercules, CA, USA) (1:2000 dilution) for 1 h at room temperature. Finally, immunodetection was performed using the ECL Prime Western Blotting Detection Reagent (GE Healthcare, Chicago, IL, USA) on an Amersham Imager 600 (GE Healthcare, Chicago, IL, USA) at the Soonchunhyang Biomedical Research Core-Facility of the KBSI.

### 4.15. Barium Chloride (BaCl_2_)-Induced Muscle Injury Model

All animal experimental procedures were performed in accordance with the protocols for animal handling and ethical standards approved by Institutional Animal Care and Use Committee (IACUC) at the Soonchunhyang University (protocol number: SCH17-0033). To induce muscle injury, 8-week-old C57BL/6J mice were anesthetized with 1% isoflurane inhalation, and the TA muscle was injured via intramuscular injection of 30 µL 1.2% *w/v* barium chloride (BaCl_2_, cat# 449644, Sigma Aldrich, St. Louis, MO, USA). At 1, 3, 5, 7, and 14 days post-injury, the TA muscles were harvested for further histochemistry and qPCR analyses. 

### 4.16. Statistical Analysis

All values are presented as the mean ± standard deviation of three biological replicates for each group, and statistical significance was assessed via one-way analysis of variance (ANOVA) with Tukey’s multiple comparison test using GraphPad Prism 9.0 (San Diego, CA, USA) software. A *p* value less than 0.05 was considered statistically significant (* *p* < 0.05; ** *p* < 0.01; *** *p* < 0.001).

## Figures and Tables

**Table 1:** Top diseases and cellular functions significantly changed in cells cultured on GelMA-PSS hydrogels, when compared to those cultured on TCP and GelMA. Diseases and biofunctions are sorted by the activation of z-score (z-score of >2).

Diseases or Biofunctions Annotation	*p*-Value	Activation z-Score	# Molecules
Motor dysfunction or movement disorder	0.00191	3.848	91
Apoptosis	0.00686	2.865	255
Formation of muscle cells	0.000459	−3.162	27
Quantity of muscle	0.000396	−3.148	28
Cell–cell contact	1.32 × 10^−10^	−3.123	105
Quantity of muscle cells	0.00239	−3.119	24
Assembly of intercellular junctions	0.0000109	−3.05	44
Formation of intercellular junctions	0.00000317	−2.936	47
Aggregation of cells	0.00116	−2.84	38
Formation of myofibrils	0.00716	−2.646	11
Quantity of striated muscle	0.00242	−2.578	15
Contractility of muscle	0.000398	−2.324	36
Microtubule dynamics	0.001	−2.098	138

**Table 2:** List of primers used for quantitative PCR.

Gene	Primer Sequence (5’ to 3’)
*B2M*	F-ACCGGCCTGTATGCTATCCAG
	R-AATGTGAGGCGGGTGGAACTG
*Myf5*	F-CTGCTGTTCTTTCGGGACCA
	R-TATTACAGCCTGCCGGGACA
*MyoG*	F-CCTACAGACGCCCACAATC
	R-CCCAGGCTGACAGACAATC
*MyHC*	F-ACGCCATCAGGCTCAAGAAGAAGA
	R-TGAGTGTCCTTGAGGATGCCTTGT
*MyoD*	F-CCGCCTGAGCAAAGTGAATG
	R-GCGGTCCAGGTGCGTAGAA
*Klf4*	F-GCCAACTACCCTCCTTTCCTG
	R-TCTTTGGCTTGGGCTCCTC
*Npnt*	F-GGCCAAACAAGTGCAAATGTC
	R-GGTGGAAGGACTCATCTTGGTT
*Ccl5*	F-GCTGCTTTGCCTACCTCTCC
	R-TCGAGTGACAAACACGACTGC
*Cxcl1*	F-CTGGGATTCACCTCAAGAACATC
	R-CAGGGTCAAGGCAAGCCTC
*Igf1*	F-TCTCACTGAAGCCAGCTCTCT
	R-CAGGCCCAGAAGCATGACA
*Pik3r1*	F-ACACCACGGTTTGGACTATGG
	R-GGCTACAGTAGTGGGCTTGG
*mTOR*	F-ACCGGCACACATTTGAAGAAG
	R-CTCGTTGAGGATCAGCAAGG
*Cav3*	F-GGATCTGGAAGCTCGGATCAT
	R-TCCGCAATCACGTCTTCAAAAT
*Lrp5*	F-GAAAGCACAATGGGTCCTCCA
	R-CTGACGCCTGTTCCACTTCT
*Axin2*	F-TGACTCTCCTTCCAGATCCCA
	R-TGCCCACACTAGGCTGACA

## Data Availability

The RNA sequencing datasets generated for this study can be found in the NCBI Gene Expression Omnibus (GSE165480).

## Abbreviations

ANOVA	analysis of variance
APS	ammonium persulfate
bFGF	basic fibroblast growth factor
BP	biological process
BSA	bovine serum albumin
CC	cellular component
DEG	differentially expressed gene
DW	distilled water
FAK	focal adhesion kinase
FITC	fluorescein isothiocyanate
FPKM	fragments per kilobase of transcript per million mapped reads
FTIR	Fourier transform infrared spectroscopy
GelMA	methacrylated gelatin
GO	gene ontology
HPSCs	human pluripotent stem cells
HSPG	heparan sulfate proteoglycans
IPA	Ingenuity Pathway Analysis
IRS1	insulin receptor substrate 1
KEGG	Kyoto Encyclopedia of Genes and Genomes
MAPK	mitogen-activated extracellular regulated signaling kinase
MF	molecular function
mTOR	mechanistic target of rapamycin
Myf5	myogenic factor 5
MyHC	myosin heavy chain
MyoG	myogenin
NMR	nuclear magnetic resonance
OXPHOS	oxidative phosphorylation
PBS	phosphate buffered saline
PFA	paraformaldehyde
PSS	poly(sodium-4-styrenesulfonate)
PVDF	polyvinylidene fluoride
SDS	sodium dodecyl sulfate
skMSC	skeletal muscle stem cell
TA	tibialis anterior
TBS	tris-buffered saline
TCP	tissue culture plate
TEMED	*N,N,N′,N′*-tetramethylethylenediamine
